# piRNAs as Modulators of Disease Pathogenesis

**DOI:** 10.3390/ijms22052373

**Published:** 2021-02-27

**Authors:** Kayla J. Rayford, Ayorinde Cooley, Jelonia T. Rumph, Ashutosh Arun, Girish Rachakonda, Fernando Villalta, Maria F. Lima, Siddharth Pratap, Smita Misra, Pius N. Nde

**Affiliations:** 1 Department of Microbiology, Immunology and Physiology, Meharry Medical College, Nashville, TN 37208, USA; krayford@mmc.edu (K.J.R.); acooley@mmc.edu (A.C.); jrumph@mmc.edu (J.T.R.); aarun@mmc.edu (A.A.); grachakonda@mmc.edu (G.R.); fvillalta@mmc.edu (F.V.); mlima@med.cuny.edu (M.F.L.); 2 School of Graduate Studies and Research, Bioinformatics Core, Meharry Medical College, Nashville, TN 37208, USA; spratap@mmc.edu; 3 Department of Molecular and Cellular and Biomedical Sciences, School of Medicine, The City College of New York, NY 100731, USA; 4 School of Graduate Studies and Research, Meharry Medical College, Nashville, TN 37208, USA

**Keywords:** piRNAs, piRNA function, piRNAs in disease pathogenesis, sncRNA

## Abstract

Advances in understanding disease pathogenesis correlates to modifications in gene expression within different tissues and organ systems. In depth knowledge about the dysregulation of gene expression profiles is fundamental to fully uncover mechanisms in disease development and changes in host homeostasis. The body of knowledge surrounding mammalian regulatory elements, specifically regulators of chromatin structure, transcriptional and translational activation, has considerably surged within the past decade. A set of key regulators whose function still needs to be fully elucidated are small non-coding RNAs (sncRNAs). Due to their broad range of unfolding functions in the regulation of gene expression during transcription and translation, sncRNAs are becoming vital to many cellular processes. Within the past decade, a novel class of sncRNAs called PIWI-interacting RNAs (piRNAs) have been implicated in various diseases, and understanding their complete function is of vital importance. Historically, piRNAs have been shown to be indispensable in germline integrity and stem cell development. Accumulating research evidence continue to reveal the many arms of piRNA function. Although piRNA function and biogenesis has been extensively studied in *Drosophila*, it is thought that they play similar roles in vertebrate species, including humans. Compounding evidence suggests that piRNAs encompass a wider functional range than small interfering RNAs (siRNAs) and microRNAs (miRNAs), which have been studied more in terms of cellular homeostasis and disease. This review aims to summarize contemporary knowledge regarding biogenesis, and homeostatic function of piRNAs and their emerging roles in the development of pathologies related to cardiomyopathies, cancer, and infectious diseases.

## 1. Introduction

Understanding the interplay between noncoding and coding RNAs represents a fast-emerging field in biomedical research. Noncoding RNAs are classified as either long non-coding RNAs (lncRNAs), which are usually longer than 200 nucleotides (nt) and small non-coding RNAs (sncRNAs) that are anywhere between 18–35 nt in length. The sncRNA that are currently known are classified into three main groups: small interfering RNAs (siRNAs, about 21 nt), microRNAs (miRNAs, about 22 nt), and Piwi-interacting RNAs (piRNAs, 24–32 nt in length) [1,2,3]. Although siRNAs and miRNAs have been studied more extensively, piRNA biogenesis and function has not been investigated in many species [4,5,6,7,8,9,10,11,12]. However, most of the knowledge about piRNA the biogenesis and function come from studies in *Drosophila*. Currently, piRNAs are thought to be the most abundant sncRNA within the genome, with over 30,000 members, excluding growing numbers of piRNA isoforms recently discovered across other species [13,14]. Characterization of piRNAs is dependent upon the binding with PIWI subfamily of Argonaute AGO/PIWI proteins. Argonaute family proteins are RNA binding proteins containing the highly conserved Piwi-Argonaute-Zwille domain (PAZ), the C-terminal PIWI domains, the less conserved N-terminal (N) and Middle (Mid) domains [15]. The PAZ domain functions as an RNA binding motif, and the PIWI domain retains RNase H fold domain, homologous to RNase H, an endonuclease [16]. Thus, Argonaute proteins are divided into two categories: AGO subfamily and PIWI subfamily. The PIWI subfamily proteins have been shown to be expressed within the germline and adult stem cells, while the AGO subfamily of proteins is ubiquitously expressed in most tissues. However, the literature shows that PIWI subfamily of proteins are also expressed in somatic cells, suggesting that piRNAs may exhibit roles outside of germline integrity maintenance [17,18]. PIWI family proteins have also been identified in mice and humans as MIWI and HIWI subfamilies, respectively [19,20]. The piRNA/Piwi complex interacts with various proteins that facilitate many functions, including: piRNA biogenesis, transposon silencing, chromatin remodeling, transcriptional and translational regulation [21]. Within the past decade, increasing number of reports implicate piRNAs as modulators of various diseases including cardiomyopathies, cancer, and infectious diseases (Table 1).

## 2. Structure and Function of piRNAs

### 2.1. piRNA Structure and Biogenesis

P-element induced wimpy testis interacting RNAs (piRNAs) are the most recently characterized class of small noncoding RNAs (sncRNAs). These RNA molecules are found in germline and somatic cells. Their sizes range anywhere from 24–31 nucleotides long and many reports identify a 5′ terminal uridine or tenth adenosine [38]. These sncRNAs have a unique feature in which a 2′ O-methyl group on the 3′ ribose is required for maturation [39,40]. This 2′ O-methyl group addition is performed by the piRNA methyltransferase Hen1 (HENMT1), a step that is conserved in all organisms [40]. Loss of *Henmt1* caused piRNA instability via decreased piRNA abundance and size, as illustrated by Lim et al. [41]. Mature piRNAs interact with a specific subset of argonaute proteins called p -element induced wimpy testis (PIWI) proteins; these RNA binding proteins interact with piRNAs, forming a ribonucleoprotein complex known as the piRNA induced silencing complex (piRISC). The piRISC is guided by piRNAs to recognize sequences at the chromatin, transcriptional, and post-transcriptional levels. Proteins containing Tudor domains (TDRDs) also associate with PIWI proteins and have been shown to play an active role in the piRNA metabolic pathway. TDRDs expedite piRISC action as well as bolster the stability of PIWI proteins by acting as a molecular scaffold [42].

Biogenesis of sncRNA generally occurs in a Dicer/Drosha dependent manner, in which double-stranded RNA precursor molecules are remodeled into functional sncRNAs [43]. Alternatively, piRNAs participate in a distinct process known as primary and secondary piRNA biogenesis [21]. This process, which has been partially described and characterized in *Drosophila*, is also conserved in *C. elegans*. The long, single-stranded piRNA precursor molecules are derived from the transcripts of protein coding genes, transposons, tRNA, rRNA, and intergenic loci [44,45,46,47] which possibly contributes to the targeted sequence specificity observed in mature piRNAs. piRNAs mostly originate from repetitive sequences within the genome including transposable elements (TEs), which can be distributed in clusters throughout the chromosomes on both DNA strands [4].

**Figure 1:**
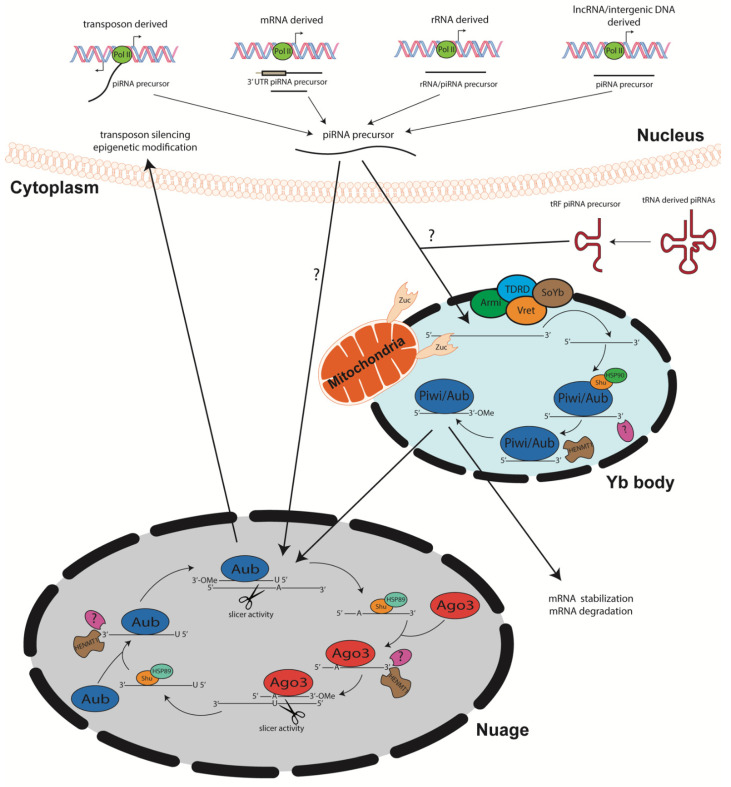
piRNA biogenesis Precursor piRNAs within the nuclear compartment are generated from sense and antisense transposon sequences, 3′UTR of messenger RNAs (mRNAs), ribosomal RNAs (rRNAs), long noncoding RNAs (lncRNAs) and intergenic DNA sequences. Furthermore, piRNAs can be derived from transfer RNA (tRNA), specifically transfer RNA related fragments (tRFs) that are processed in the cytoplasm. During primary biogenesis within the Yb body (shown to occur in somatic cells) precursor piRNA is associated with various processing proteins including Tutor domain containing protein (TDRD), Armitage (Armi), Vreteno (Vret), and Sister of Yb (SoYb), though their specific functions during piRNA biogenesis is not well understood. piRNA precursors are processed by mitochondria membrane bound protein Zucchini (Zuc) into small segments. Shutdown (Shu) and Heat shock protein 90 (HSP90) shuttle piRNAs onto Piwi proteins. An unknown endonuclease further processes the 3′ end of the piRNA before piRNA methyltransferase Hen1 (HENMT1) adds a 3′-OMe group. Mature piRNAs enter the ping pong cycle to produce progeny piRNA, re-enter the nucleus for transposon silencing and epigenetic modification, or enter the cytoplasm to play roles in mRNA transcript stabilization or degradation. The ping-pong cycle occurs in the Nuage (shown to occur in germ cells), where antisense piRNAs bound to Aubergine (Aub) can bind to sense piRNA precursor molecules, allowing the RNAse H-like slicer activity to process the precursor piRNA into a smaller segment. Shu and HSP89 shuttle processed piRNAs onto Argonaute 3 (Ago3). An unknown protein processes the 3′ end, and subsequently HENMT1 adds the characteristic 3′-OMe that is required for piRNA stabilization. Mature, sense, Ago3 bound piRNAs can continue to process more antisense precursor piRNAs that will bind to Aub and continue the cycle of piRNA secondary biogenesis. The mature, stable piRNAs synthesized from the ping-pong cycle can then enact various piRNAs function in the nucleus and/or in the cytoplasm.

Primary piRNA biogenesis begins with transcription of single stranded piRNA progenitor molecules by RNA polymerase II, *albeit* recruitment mechanisms and promoter regions remain elusive [48] constituting new areas of research. Processing of these precursors operates in a Dicer/Drosha independent manner. The single stranded piRNA precursor molecules are shuttled out of the nucleus into Yb bodies, cytoplasmic membranesless organelles [49] (Figure 1). In the Yb bodies, the precursor piRNA/Piwi complex interacts with TDRD, Armitage, Vreteno, and Sister of Yb proteins. The function of these proteins during piRNA biogenesis remains elusive, however, they have been shown tso colocalize to Yb bodies in *Drosophila* germline, suggesting that they play instrumental role in piRNA biogenesis [50,51,52,53,54,55]. Yb bodies have been shown to be surrounded by mitochondria, facilitating the mitochondria membrane anchored endonuclease Zucchini to process precursor piRNAs into smaller segments [51,56]. With the help of Shutdown (Shu) and Heat shock protein 90 (Hsp90), piRNAs then complex with Piwi proteins [57,58]. Lastly, the 3′ end is processed by an unknown protein, after which the HENMT1 adds a methyl group to the 2′ carbon of the ribose on the 3′ end of the transcript [40,59]. This modification is crucial for piRNA stability [41]. Mature piRNA complexes can either reenter the nucleus to induce transposon silencing and gene regulation or initiate secondary piRNA biogenesis, also known as the ping pong cycle [4,60]. Interestingly, different piRNA subsets have been shown to complex with different PIWI proteins. Piwi and Aubergine (Aub) have a propensity for piRNA sequences that are specifically antisense to transposons with a 5′ uridine. In contrast, Ago3 proteins bind to sense piRNAs containing adenine at the 10th nt position, with no preference for 5′ U piRNA sequences [4,7,44,61] (Figure 1).

The ping-pong cycle which has been the major contributor of piRNA biogenesis, depends on Aub and Ago3 associated piRNAs. Once long, single stranded precursor piRNAs are transcribed via RNA polymerase II, they are shuttled to the nuage, an electron-dense phase separated granules anchored to the cytoplasmic face of the nuclear pore [60] TDRD proteins may serve as scaffolds and initiators of ping pong cycle, as mutations within these proteins disrupt piRNA biogenesis [56,62,63,64]. The amplification cycle involving these PIWI proteins calls for mature 5′ U piRNAs complexed with Aub to recognize specific complementary sequences within the transcript. Once complementarity between the U:A nt is achieved, the Aub enacts endonucleolytic cleavage of the target piRNA at the 10th/11th nt of the sequence. This slicing activity generates the 5′ end of a new sense piRNA, including a 10 nt long 5′ overlap with the initial antisense piRNA and an adenosine residue at the 10th nt. Shu and Hsp89 are hypothesized to load the newly generated piRNA onto Ago3 [57,58]. Subsequent trimming by an unidentified enzyme and 3′ modification by piRNA methyltransferase finalizes piRNA maturation. The newly formed complex then produces more antisense Aub-bound piRNAs from piRNA clusters within the transcripts via the same biogenesis mechanism. The secondary piRNA amplification of the ping pong cycle has been linked to targeted post-transcriptional gene silencing [4,61]. piRNA biogenesis from sense/antisense transposons has been defined but requires further investigation [65]. However, others have shown that piRNAs derived from both mRNA and lncRNA are typically generated from the 3′ UTR but the mechanism is yet to be fully unraveled. Noncanonical by-products of concurrent transcription of adjacent genes are also thought to be piRNA precursor transcripts, which hail from dual-stranded clusters [66]. Though much of the piRNA biogenesis pathway is conserved amongst most organisms [67], further studies are needed to fully elucidate mechanisms within the pathway (Figure 1).

### 2.2. Transposon Silencing

One of the earliest functions of piRNAs is their ability to serve as regulators of TE movement throughout the genome. TEs can be randomly inserted throughout the genome, causing changes in protein coding genes and regulatory sequences, which can affect gene expression and cause the production of defective or malfunctioning proteins [68]. It is well documented that piRNAs are vital to ensuring genomic integrity, as the piRNAs/Piwi complex can monitor TE activity by silencing TEs post transcriptionally [69]. Mutations within the germline have been shown to cause an increase in retrotransposons, which causes the death of germ cells in conjunction with various deficiencies within microtubule cytoskeletal polarization. Consequently, alterations in this conserved mechanism leads to changes in polarized localization of specific proteins and mRNAs that are necessary for oogenesis in *Drosophila* [70].

Sarot et al. illustrated that Piwi is required for silencing transposons within the *3am* locus in gonadal somatic cells, which is a well-known active region [71]. A vast number of piRNAs originate from transposon regions of the genome, as shown by others and recently our group [37,65,72,73]. Several studies have demonstrated the importance of PIWI proteins in the regulation of transposon silencing in *Drosophila*. For example, *aub*-mutants illustrated increased transposon activity [70,74,75,76,77]. Similarly, Ago3 and Aub are known to colocalize in the nuage for secondary piRNA biogenesis; mutations within either of these proteins triggered defects in biogenesis and elevated transposon transcripts [78]. The vital function of piRNAs in maintaining genome stability has been illustrated using knockout models designed to understand the roles of PIWI proteins [8,79]. In germ cells of *mili/miwi2* KO mice, Carmell et al. observed a significant increase in transposable elements [79]. Transposon silencing remains a critical function of piRNAs, however further studies should aim to describe the targeted action of piRNAs as well uncover other mechanisms that can contribute to this silencing.

### 2.3. Epigenetic Regulation

piRNAs have been shown to participate in epigenetic regulation through two mechanisms: activation of sequence specific de novo methylation and chromatin remodeling. Huang et al. demonstrated a vital role of the piRNA/Piwi interface in epigenetic programing in *Drosophila*. Once piRNA complementary sequences were introduced in ectopic sites, several DNA binding proteins, including Piwi and heterochromatin protein 1 (HP1a), induced increased H3K9me2/3 recruitment, which ultimately led to reduced RNA polymerase II enlistment. This finding supports the hypothesis that piRNAs are necessary for the recruitment of epigenetic associated proteins to precise genomic regions/locations in *Drosophila* [80,81]. Deletion of PIWI proteins in murine models significantly altered the epigenetic makeup throughout the genome [8,79]. Watanabe et al. demonstrated simultaneous coactivation of piRNA-mediated DNA methylation and transcription of piRNA dependent regions in mice. Retrotransposons in piRNA dependent regions and in piRNA clusters were knocked out in a mouse model. The deleted regions were shown to play a role in the initiation of piRNA-mediated methylation. These results showed the importance of MIWI in determining chromatin structure through direct targeting of piRNAs to genomic regions, which also has been observed in other organisms [82].

The synergic interactions between methyltransferases, histone modifying proteins, chromatin remodeling proteins, and piRNA/Piwi complex is implicated in de novo methylation and subsequent suppression of TEs within the germline. However, the mechanism is not well understood and necessitates further investigation. In *Drosophila*, Piwi has been shown to interact with chromosomes in somatic tissues, implicating its role in inducing epigenetic modifications at various binding sites [81,83,84]. A study showed that a piRNA/Piwi complex in *Aplysia* nerve cells promoted methylation of CpG islands within the promoter region of transcription factor CREB2, further solidifying the role of piRNA/PIWI complex in epigenetic gene regulation [85,86]. piRNA/Piwi complexes can also recruit DNA methyltransferases to target CpG sites in non-TE protein coding regions, which alters transcriptional activity in mice [87]. Fu et al. demonstrated that genomic regions neighboring differentially methylated CpG areas were fortified with sequence matches to transfected piRNAs in somatic cancer cell lines [88]. This transcriptional repression can then possibly be inherited at the target sites in mammals, however further research is needed to elucidate this fully. For example, overexpression of piR-021285 induced methylation of *ARHGAP11A* at CpG site within the 5′ UTR/first exon, which dampened messenger RNA expression while simultaneously inhibiting breast cancer programmed cell death [28]. Recent efforts to uncover mechanistic machinery of piRNA directed DNA methylation in mammals have identified proteins essential to this function. SPOCD1 has recently been identified as a MIWI2 associated protein that is required for TE silencing via piRNA mediated methylation. Loss of *Spocd1* induced infertility in male mice without altering piRNA biogenesis or MIWI2 nuclear localization [89]. Similarly, recruitment of TEX15 has been shown to be an executor of piRNA directed methylation, as TEX15 is required for the nuclear function of MIWI2 [90]. Further research should delineate exact mechanisms that trigger and induce piRNA/Piwi dependent methylation throughout the genome. Inheritance of these piRNA induced epigenetic modifications should also be explored in depth.

Although the mechanism of piRNAs induce methylation of genomic regions is unclear, studies have provided evidence indicating that piRNAs serve as guides for histone modifying and chromatin remodeling proteins. Mohn et al. utilized chromatin immunoprecipitation to suggest that the ribonucleoprotein complex targets sequences within euchromatin via preliminary transcripts, while also targeting heterochromatin via RNA/DNA interaction. This is a direct result of piRNA sequence complementarity, illustrating the wide yet potentially specific target [18,91]. Piwi has been found to interact with HP1a directly, and colocalization of these proteins has been observed on many sites throughout chromosomes in *Drosophila* [80,81]. As regulators of the position effect variegation (PEV), which decreases gene expression of euchromatin genes near heterochromatin sites, Piwi and Aub can silence gene expression via heterochromatin assembly [83]. piRNA dependent recruitment of histone modifying proteins remains a mystery, however current research should continue to decode this mechanism.

### 2.4. Post-transcriptional and Translational Control

In several organisms, it has been shown that 3′UTRs of protein coding mRNAs are sources for piRNA production in *Drosophila*, mice, and Xenopus, indicating that piRNAs also can regulate mRNA turnover [92]. It was observed that VAS protein levels increased in *aub* and *ago3* mutants, which could possibly be attributed to decreased number of piRNAs derived from *vas* mRNA [78,93]. Miwi proteins in mice have been shown to complex with and stabilize mRNAs of genes necessary for post-meiotic steps of spermatogenesis [94]. Lee et al. showed that piRNAs could target non-transposon genes that contribute to the regulation of spinal shape [95].

In terms of piRNA/mRNA interaction, piRNAs require base pairing at both the 2–11 nt at the 5′ end of the piRNA as well as within the 12–21 nt of the sequence [28]. To regulate mTOR expression, piR-55490 was observed to bind to the 3′ UTR, initiating mRNA degradation and muffle lung cancer development [25]. Esposito et al. concluded that testis and brain specific piRNAs can regulate *MTNR1A* expression in human somatic cells [96]. piRNAs have also been shown to play a role in mRNA decay via deadenylation of the 3′ end of mRNA transcripts. In *Drosophila*, piRNA dependent degradation of maternal mRNA is dependent upon Smg, deadenylase CCR4, and the piRNA/Piwi complex. Impaired piRNA regulation induced transcript stabilization [97,98]. The post-transcriptional/translational regulatory role of piRNAs outside of the germ line has yet to be fully understood, however these studies strongly implicate piRNAs as critical post-transcriptional regulators.

Evidence of chromatin remodeling, de novo methylation, and direct transcriptional regulation shows the multifaceted roles of piRNAs [28,37,68,69,70,71,72,73,74,75,76,77,78,79,80,81,82,83,85,86,87,88,89,90,91]. These processes clearly classify piRNAs as regulators of gene expression in various capacities. Currently, little evidence implicates the piRNA/Piwi complex in translational regulation, however several emerging reports indicate that PIWI proteins can interact with translational proteins [99,100,101]. For example, Grivna et al. showed that Miwi binds to eIF4E, the mRNA 5′ cap binding protein essential for guidance to ribosomes in the cytoplasm and translational control [99]. A separate study reported that Mili/eIF3a complex interacts with eIF4E/eIF4G 5′ cap binding complex [101]. Recently, Ramat et al. reported that PIWI protein Aub is required for translational activation of *nanos* mRNA, which is essential in germ line survival and pluripotency. Through direct physical interaction, Aub complexes with poly(A)-binding protein (PABP) and translation initiation factor eIF3. Using polysome gradient profiling, Aub was shown to be essential for the initiation step of translation [100]. These studies suggest PIWI proteins potentially play a role in initiation and activation of translation; however, further studies are needed to completely delineate the functions of PIWI proteins and piRNAs in this process.

## 3. piRNAs vs other sncRNAs

Although piRNAs are the most newly identified class of sncRNAs, they are the least classified. Hence, several research efforts have been geared towards delineating similarities and differences between piRNAs and other sncRNAs, specifically miRNAs and siRNAs. piRNAs are widely and differentially expressed in various tissues and cell types, but mostly have been detected within the germ cells of mammals, fish, and *Drosophila* [102,103]. In terms of biogenesis, it is well understood that piRNAs diverge significantly from miRNAs and siRNAs. piRNAs are generated via RNaseIII-independent pathways and do not involve dsRNA progenitors. Since these unique sncRNAs are generated from long single stranded precursors, they show preferential cleavage at uridine residues and then complex with PIWI proteins [65]. In contrast, siRNAs are processed by Dicer from small dsRNA complexes with a distinct 2 nt 3′ overhang and 5′ phosphate group. miRNAs are transcribed via RNA polymerase II from primary miRNA (pri-miRNA) precursors which contain stem loop structure. These molecules undergo further processing that give rise to classical miRNAs [104,105].

Structurally, miRNAs are short, single stranded noncoding RNAs, generally 20–24 nucleotides in length, which are encoded in eukaryotic cells and function in various signaling pathways [106,107]. siRNAs, however, are 21–23 nt long RNA duplexes that interact and activate the RNA-induced silencing complex (RISC) [108,109]. Functionally, miRNAs are recognized for control of mRNA stability and translation. These sncRNAs are found within the cytoplasm where they recognize and bind the 3′ UTR of target mRNA complementary sequences via the miRNA “seed sequence” located at their 5′ end [110]. The consequence of this interaction is either mRNA degradation or translational repression. In contrast, siRNAs must accomplish 100% complementarity with mRNA target seqence in order to enact repressive function [109].Though piRNAs and miRNAs can bind to various target molecules, siRNAs are specific to one mRNA target and regulate expression exclusively through endonucleolytic cleavage via RISC [109]. miRNAs are categorized via sequence conservation and the seed sequence, meaning many miRNAs within the same family share similar targets in intertwining signaling pathways [111]. Current research reveals untraditional roles of miRNAs within the nucleus, including transcriptional silencing/activation, and alternative gene splicing [112,113,114]. We also recently showed the role of miRNA in translation initiation [115]. Although the mechanisms which activate these noncanonical roles remain to be fully understood, several debates regarding miRNA nuclear translocation persist [114]. Several studies have reported that miRNAs activate gene expression via promoter region binding by utilizing sequence complementarity [112,116].

In contrast, piRNAs are about 24-31 nucleotides long and were discovered in *Drosophila* mutants that underwent unequal division of stem cells within the germline. As the most abundant sncRNAs [13,14], extensive research has begun to uncover the vast roles these molecules play in cell homeostasis and disease. Developmentally, they are essential for germ line survival and enact transposon silencing, actively maintaining genome integrity. Any defects and disruptions in piRNA biogenesis is detrimental, causing germline specific cell death and sterility via upregulation of transposon expression as seen in mice and fish [117]. Other functions include conserving telomere structure, controlling RNA silencing, and inducing epigenetic factors that alter chromatin structure. More recently, studies have begun to investigate piRNA function in somatic cells, where expression seems to be regulated in a tissue specific manner. Targeting of mRNA sequences is believed to be similar to that of miRNAs, specifically utilization of the seed sequence. Unlike miRNA, Piwi/piRNA complexes can also recruit other proteins such as CCR4-NOT and Smg to create RISC like complexes called pi-RISCS, which repress mRNA translation via imperfect base pairing [118,119]

For piRNAs to enact their gene silencing function, they must complex with a specific subset of argonaute proteins called PIWI proteins. The association with Piwi and Dicer independent mechanism are the main distinguishing factors between siRNAs and piRNAs. However, similarly to other sncRNAs, piRNAs are loaded into the PIWI protein and guide argonaute to target sequences using Watson-Crick base pairing and seed sequence complementarity. When complementarity is reached, RNase H-fold activity of the PIWI domain cleaves phosphodiester bond of two nucleotides in the target RNA that pair with the 10th and 11th nt of the piRNA sequence [65]. In contrast, Dicer within RISC displays RNaseIII activity, initiating two cuts which are 21–22 nt apart on the target sequence [120].

Post transcriptional functions of piRNA are poorly understood, and only a few reports show different PIWI proteins complexing with mRNA 5′ cap and eIF proteins to regulate translation [99,101]. Extensive research is needed in this area to fully elucidate this potential function. In contrast, miRNAs and siRNAs are known mediators of post transcriptional regulation, and the pathways they regulate differ according to cell type, organism, and argonaute protein binding. Argonaute proteins complexed with miRNAs or siRNAs utilize slicer activity and/or slicer dependent mechanisms to employ repression functionality. This includes inhibition of eIF4E cap binding and mobilization of other proteins that participate in translational repression and mRNA degradation [98,121]. High complementarity is required for target mRNA degradation via slicing, mediated by degradation proteins such as XRN1, exosomes and Ski complexes [122]. Translationally repressed RNAs can be degraded through deadenylation, decapping, and subsequent degradation in P-bodies [97,98]. Though many differences reside in biogenesis and many similarities reside in function of sncRNAs, further studies are required to identify the unique roles that each of these RNAs play in the regulation of gene expression.

## 4. The Role of piRNAs in Cardiomyopathies

It was previously thought that piRNAs expression was exclusive to germ cell lines, but recent studies have shown that piRNAs are also expressed in non-germline cells [37,123]. In recent times, it has been theorized that piRNAs are expressed in higher eukaryotic genomes. This theory came from the discovery that piRNAs regulate transposons and higher eukaryotes house large repositories of transposable elements. Recent advances indicate that piRNAs are found in the central nervous system, liver, cardiovascular system, and in circulating serum exosomes [35,124]. Researchers have recently taken interest in the role of piRNA expression in the differentiation of cardiomyocytes.

Greca et al. sought to investigate the role of piRNAs in the development of cardiac progenitor cells. To examine this, the group analyzed RNA-seq data from H9 human embryonic stem cells, early mesoderm progenitor cells and cardiomyocytes. Stem-loop retrotranscription primers were generated to obtain cDNA from piRNAs of interest and real time PCR was carried out to quantify piRNA expression. It should be noted that mapped reads from mRNAs and other non-coding RNAs were filtered out by size to accurately determine the role of piRNAs in the development of cardiac progenitor cells. The group reported that there are at least 447 piRNAs involved in the progression of pluripotent stem cells to cardiomyocytes. Furthermore, cardiac progenitor cells expressed increased levels of mitochondrial tRNA/rRNA derived piRNAs, followed by a significant upregulation in HIWI2 expressing cells [123].

Since the majority of these piRNAs were derived from other types of RNAs, the group noted that further validation must be performed to ensure their accurate classification. However, approximately 90 of the 447 piRNAs involved in the development of cardiomyocytes originated from the mitochondrial genome [110]. It should be noted that piRNAs were found to be uniformly scattered throughout the genome, aside from the mitochondrial genome where they were expressed in clusters. RNA-seq data suggested that nuclear encoded lncRNA *MALAT1* and *TTN* encompass the highest number of piRNAs in cardiac progenitor cells. RNA-seq data demonstrated that *MALAT1*-derived piRNAs include piR-4403262, and piR-4424378. *TTN*-derived piRNAs include piR-1551388, piR-4193743, and piR-2715002. Interestingly, the group reported that six times more piRNAs were downregulated in cardiac progenitor cells compared to pluripotent stem cells. This study showed that piRNAs are involved in cardiac cell differentiation and maturation, thus dysregulation of piRNA can result into cardiac pathology [123].

Vella et al. also investigated the role of the piRNome, the global expression profile of piRNAs within the cellular system, in different cardiac cell types using biopsies from patients undergoing heart surgery. These biopsies were used to isolate cardiac progenitor cells, which were grown as undifferentiated self-adherent 3D cultures in suspension (cardiospheres) or as adherent monolayers on a plate (cardiosphere-derived cells). They also isolated cardiac fibroblasts from these biopsies and examined the piRNome among each of these cell types via microarray analysis and RT-PCR validation. The group reported that 15,311 piRNAs were expressed in all three cell types, among the 23,000 piRNAs within the dataset used for the study. Of the identified piRNAs, 641 were upregulated and 1,381 were downregulated in cardiospheres compared to cardiosphere-derived cells. However, there were only 255 upregulated and 780 downregulated piRNAs in cardiospheres compared to cardiac fibroblasts. Furthermore, when cardiosphere-derived cells were compared to cardiac fibroblasts, only 52 piRNAs were upregulated and 129 were downregulated. These findings suggest that piRNAs are differentially expressed in cardiac cells and have distinct functions in individual cell types. To further support this theory, the group reported that certain piRNAs such as DQ570326 and DQ58246 are expressed at higher levels in cardiosphere-derived cells compared to cardiospheres. Furthermore, some piRNAs, such as DQ579896 and DQ581624 are upregulated in cardiac fibroblast but not cardiospheres or cardiosphere-derived cells [125].

Di Giacomo et al. reported that the inhibition of LINE-1 retrotransposons was mediated by piRNAs [126], and this mechanism was shown to attenuate ischemic heart disease via piRNA-mediated activation of AKT [127]. Vella et al. examined LINE targets of upregulated piRNAs in cardiospheres, cardiosphere-derived cells and cardiac fibroblasts and they found that the upregulated piRNAs targeted all three classes of LINE reverse transcriptase: LINE-1, LINE-2 and CR1. More specifically, the transcript levels of LINE-1 transcripts were lower in cardiospheres and cardiosphere-derived cells compared to cardiac fibroblasts. This corresponded to increased expression of phosphorylated AKT in cardiospheres and cardiac derived cells compared to cardiac fibroblast. It was also demonstrated that piRNA-mediated phosphorylation of the Ser473 residue of AKT was associated with the phosphorylation of the Ser9 residue of GSK3β to inactivate GSK3β’s kinase activity in cardiospheres and cardiosphere derived cells. Hence, piRNA expression is associated with cardiac regeneration and mediated by AKT-mediated phosphorylation. However, it is possible that cardiac piRNA expression has downstream effects on cellular signaling pathways that involve GSK3β, which is a known repressor of cardiac hypertrophy by phosphorylating proteins involved in cell proliferation such as β-catenin and NFATC4 [128]. Overall, this study showed that the piRNome may play a role in regeneration within cardiac progenitor cells—indicating that piRNAs may be involved in cardiac pathogenesis and may serve as therapeutic targets [125].

In 2020, Gao et al. investigated the potential role of piRNAs in cardiac hypertrophy by examining the global expression of piRNAs in mice. Samples were obtained from the left ventricle 4 weeks after a transverse aortic construction surgery, then subjected to microarray analysis. The group found that piR-141981, piR-6999 and piR-110550 were increased but piR-13375 and piR-106654 were decreased in mice that underwent a transverse aortic construction surgery versus a placebo/sham surgery. Of these piRNAs, piR-141981, piR-110550 and piR-13375 increased mRNA expression of β-myosin heavy chain, a cardiac stress marker and biomarker of cardiac hypertrophy. The group also examined the global expression of these three piRNAs and found that piR-141981 was expressed at higher levels in the heart, compared to other organs [22].

Since piR-141981 was previously unclassified, the group named it the cardiac-hypertrophy-associated piRNA (CHAPIR). CHAPIR was found to be expressed at higher levels in cardiomyocytes compared to cardiac fibroblasts. In mice, CHAPIR is located on chromosome 4 within intron 1 of *Gm12648*. The group generated CHAPIR knockout mice to observe the role of this piRNA in the development of hypertrophy following traverse aortic construction surgery. They found that a deficiency of CHAPIR blocked the development of cardiac hypertrophy following surgery. This was mediated through the attenuation of hypertrophic biomarkers including the expression of *Anp*, *Myh7* and *Bnp*. It should be noted that *Anp* and *Bnp* encode atrial and brain natriuretic protein, respectively. *Myh7* encodes the heavy β-myosin chain [22]. The group found that CHAPIR increases the stability of *Parp10* mRNA transcripts by inhibiting METTL3-mediated N6-methyladenosine methylation of *Parp10* mRNA. More specifically, *Parp10* gene expression is upregulated when the CHAPIR-PIWIL4 complex interacts with METTL3, blocking N6-methyladenosine (m6A) methylation of Parp10 mRNA transcripts. Under “normal” conditions, m6A destabilizes *Parp10* mRNA transcripts and accelerates their degradation. However, in the presence of CHAPIR, *Parp10* mRNA is translated into PARP10, which inactivates GSK3β through mono-ADP-ribosylation. Inhibition of GSK3β kinase activity then leads to the onset of hypertrophy, marked by nuclear accumulation of NFATC4. Under “normal” conditions NFATC4 is phosphorylated by activated GSK3β—promoting its nuclear export and decreased hypertrophic response. However, when GSK3β’s kinase activity is inhibited it cannot phosphorylate NFATC4, which remains in the nucleus—promoting cardiac hypertrophy. This finding supports the theory that piRNA activity may be associated with cardiomyopathies since cardiac progenitor cells do not express large amounts of piRNAs under normal conditions [22].

Furthermore, Yang et al. demonstrated that exosomal piRNAs have the potential to serve as biomarkers in patients with heart failure. The group isolated exosomes from the blood of heart failure patients and age matched controls. RNA was isolated from the exosomes and the expression of piRNAs was examined using RNA-seq. They reported that approximately 585 piRNAs were upregulated and 4,623 were downregulated in the exosomes of patients with heart failure. Furthermore, has-piR-006426 and has-piR-0200009 were among the most downregulated piRNAs derived from the serum exosomes of heart failure patients. These findings indicate that hsa-piR-006426 which is located on chromosome 17p11.2 and has-piR-0200009, which is located on chromosome 7q35 may be involved in heart failure and may serve as clinically relevant biomarkers for heart disease [23]. In a separate study, Rajan et al. discovered that PiR_2106027 was elevated in the serum of patients with Troponin-I negative myocardial infarctions and downregulated in healthy patients. Hence, PiR_2106027 may serve as a biomarker for myocardial infarctions [24]. Furthermore, recent studies suggest a correlation between piRNAs, mTOR and cardiac tissue regeneration. Specifically, piR-55490 has been shown to regulate mTOR, which consequently promotes angiogenesis and cardiac tissue regeneration. This suggest that piRNAs may regulate both cancer and cardiovascular disease/regeneration in an mTOR-dependent manner [25,26].

Overall, these studies suggest that piRNA expression differs among cardiac progenitor cells and cardiac fibroblasts, suggesting that piRNAs may play distinct roles in different types of cells. Furthermore, cardiac progenitor cells tend to express downregulated levels of piRNAs compared to other cardiac cells such as cardiac fibroblasts. However, the dysregulation of specific piRNAs, such as CHAPIR promotes the development of cardiomyopathies, including cardiac hypertrophy. Furthermore, other piRNAs such as has-piR-0200009 may serve as clinically relevant biomarkers of cardiomyopathies and heart failure. Although the exact function of piRNAs in the progression of cardiomyopathies remain to be fully elucidated, current research suggest that piRNAs may serve as therapeutic targets and clinically relevant biomarkers.

## 5. Influence of piRNAs in Cancer Pathogenesis

It has been found that PIWI proteins in human and mice, such as PIWIL1, PIWIL2, PIWIL2 proteins, and HIWI are expressed in various types of tumor cells [129,130]. PIWIL1 overexpression is associated with cell cycle control and proliferation [130] while PIWI2 has roles in anti-apoptotic signaling [131]. Dysregulated expression of piRNAs has been reported in human cancers, including gastric, bladder, breast, colorectal and lung cancer. These findings indicate that the piRNA dysregulation may be linked to cancer development, onset and progression. The potential role of piRNAs in cancer has just emerged and requires investigation. Although the functional roles of specific piRNAs is poorly understood in human cancer, the following findings implicate piRNA mediated regulation as an important focus for cancer research.

### 5.1. Breast Cancer

Breast cancer is one of the most common types of cancers and is second only to lung cancer for cancer related deaths in women. Numerous piRNAs have been found to play a role in this disease. Analysis of RNA sequences from breast cancer cells *versus* normal tissues showed differential expression of piR-4987, piR-20485, piR-20582, and piR-20365. Of these, upregulated piR-4987 was associated with lymph node metastasis of breast cancer [28]. Another study found that piR-021285 can methylate several genes related to breast cancer in cell culture studies [27]. Anti-cancer effects were also observed with piRNA. For example, piR-36712 was shown to exhibit tumor suppressor effects by increasing the efficacy of paclitaxel and doxorubicin using animal model of breast cancer [132]. piR-DQ598677 is downregulated in cancer. When overexpressed, breast cancer growth is inhibited through increased degradation of mRNA for TAX1BP, TNFESF10B, and SFRP2 which have preestablished roles in tumorigenesis [27,28,132,133].

### 5.2. Lung Cancer

Lung cancer is the number one cause of cancer-related death worldwide. One study demonstrated that in human bronchial epithelial cells, piR-L-163 was upregulated upon cisplatin treatment *in vitro* and *in vivo*. This piRNA is capable of regulating ezrin-radixin-moesin (ERM) proteins which have altered expression during cancer progression [29]. piR-651 can act as an inhibitor of apoptosis in human lung cancer cells. Inhibition of this piRNA increased expression of pro-apoptotic caspases, attenuating tumor growth. When upregulated, piR-651 is believed to promote upregulation of cyclins and CDKs to increase cell proliferation. piR-55490 is capable of binding mTOR, inhibiting the protein and reducing expression of its target genes HIF-1, PGC-1α, and PPARγ. In treatment with this piRNA, a decrease in lung cancer cell proliferation was observed [25,134].

### 5.3. Colorectal Cancer

Colorectal cancer is one of the most common cancers in both men and women and the third most common cause of mortality. One research group has implicated piR-823 as a factor in colorectal cancer when they showed knocking down of this piRNA induced G1 arrest and suppressed inhibition of apoptosis in colorectal cells. It was suggested that piR-823 promotes carcinogenesis through interaction with HSF1, which can regulate heat shock protein expression and is often overexpressed in highly malignant cancers [135,136]. Colorectal cancer tumor growth and metastasis was found to be enhanced by increased levels of oncogenic piR-1245 which is capable of binding and subsequently suppressing multiple tumor suppressors such as ATF3, DUSP1, and SESN2 by degradation of mRNA. Histologically, colorectal cancer cells with elevated piR-1245 were more poorly differentiated and exhibited higher metastatic potential [31].

### 5.4. Gastric Cancer

piRNAs 823 and 651 were also shown to have a role in gastric cancer where treatment with piR-823 inhibited cancer growth *in vivo* and piR-651 overexpression was associated with metastasis. One group proposed that these piRNAs could be biomarkers for early diagnosis similar to serum carcinoembryonic antigen (CEA). This idea is a point of significant interest, as piRNA does not degrade as readily as these other makers and is easier to detect. piRNA 823 has also been associated with worse prognosis in patients with multiple myeloma, via regulation of de novo DNA methylases DNMT3A, 3B and angiogenesis [32]. The piRNA pathway has been found to be required for de novo methylation of murine *Rasgrf1* gene. Aberrant methylation of the differentially methylated region of *Rasgrf1* is associated with an increased risk of gastric cancer [137]. PIWI proteins can also regulate the progression of gastric cancer in a piRNA independent manner via UPF1 mediated nonsense mediated mRNA decay (NMD) mechanism [138].

### 5.5. Bladder Cancer

Transcript profiling of bladder cancer tissues implicated piRNA-DQ594040 as downregulated in bladder cancer. Subsequent in vitro experiments on human bladder cancer cell lines indicated that overexpression of this piRNA led to inhibition of apoptosis and colony formation, possibly through regulation of Tumor Necrosis Factor Superfamily Member 4 (TNFSF4) [33].

### 5.6. Liver Cancer

In relation to hepatocellular carcinoma (HCC), piR-Hep1 was found to be upregulated in several HCC tissues. Upon silencing of this piRNA, a decrease in cancer viability and invasion was observed. It is believed that piR-Hep1 upregulates PI3K/AKT signaling. Additionally, though its role in cancer has not been elucidated, significant elevations in piR 013306 are specific to liver cancer, meaning it could serve as a novel biomarker [33,35] which require further investigation to harness its importance in HCC.

### 5.7. Melanoma

Though no specific piRNA have been associated with melanoma, aberrations in PIWI-piRNA pathway proteins such as PIWIL3 and DCP1A are predictors of more aggressive forms of this cancer [139].

### 5.8. Glioblastoma

PIWIL3 expression has been shown to have a direct correlation with glioblastoma aggressiveness, both in terms of growth and metastasis. It was also reported that downregulation of PIWIL3 reduced progression of gastric cancer through JAK2/STAT3 signaling. piR-30188 can act as a tumor suppressor through miR-367-3p regulation. piR-8041, normally downregulated in glioblastoma, can suppress cancer growth when upregulated through regulation of MAPK signaling and heat shock protein expression. piRNAs were also seen to be of use in therapeutic delivery, where piRNA-DQ593109 promotes drug delivery in the glial tumor microenvironment [140,141,142].

### 5.9. Pancreatic Cancer

Little is known about the role of piRNA in pancreatic cancer. Currently, RNA-seq of pancreatic ductal adenocarcinoma tissue revealed significant downregulation of piR-017061, located within sno-HBII-296A. Additionally, piR-016658, piR-001311 (PV) have been detected in the extracellular RNA of pancreatic cancer patients [36].

## 6. piRNAs and Infectious Diseases

The role of piRNAs in infectious diseases research remains to be elucidated. Current piRNA studies in infectious diseases have been modeled in arthropods and nematodes. These studies indicate that infections can induce aberrant expression of TEs in somatic tissues [143,144]. This finding suggests that infections can significantly influence genome stability, host gene transcription, splicing, and/or RNA editing [73].

Small RNA-induced regulation has been reported to be the primary antiviral defense mechanism in nonvertebrate organisms [145,146]. In *Caenorhabditis elegans*, piRNAs were shown to be dysregulated in response to changes in environmental conditions and infection: increased temperature downregulated piRNA expression whereas bacterial infection led to upregulation of piRNA expression profile. These changes in piRNA expression were multigenerational, despite removal of the stimuli—suggesting epigenetic regulation [144]. In *Anopheles gambiae*, piRNAs and siRNAs were profiled and found to have increased expression under stress. The upregulated piRNAs were derived from both genic and TE-associated regions of the genome [73]. Furthermore, studies in *Drosophila* suggested a possible interplay between host siRNA and piRNA response mechanisms and that the piRNA and siRNA pathways may regulate the same RNA substrates [143]. Others suggested that the piRNA pathway works in concert with the siRNA pathway to inhibit viral replication in mosquitos [147]. Considering this intricate relationship between these small RNAs during response to infection, it becomes evident that more work is required to identify and characterize the possible synergistic interaction between these two pathways [73].

Arthropod genomes contain sequences derived from integrations of DNA and nonretroviral RNA viruses. These sequences, known as endogenous viral elements (EVEs), are acquired evolutionarily through past viral infections. Studies have implicated these EVEs as templates for the biogenesis of piRNAs in several mosquito species and cell lines [148]. Host immune response to these integrated viral elements has been found to be associated with PIWI4. PIWI4 is involved in EVE-derived piRNA maturation and preferential binding to piRNAs that are antisense to a given viral genome, which function to inhibit viral replication. For example, a study on the response of piRNAs to gammaretrovirus in koalas suggests that piRNAs mount both innate and adaptive immune response to viral genome integration [149]. These studies give critical insights that initiate uncovering the mechanisms of viral immune responses developed over time across different species.

Studies detailing the significance of piRNA in infectious diseases beyond viral infections remain limited. Recently, a group showed that piR-27283 upregulated in human skin tissues infected with *Mycobacterium leprae* could serve as a potential biomarker of disease. They further implicated piRNAs as possible determinants of macrophage function and nerve regeneration through interactions with IL6R and GAS6 [150].

**Figure 2:**
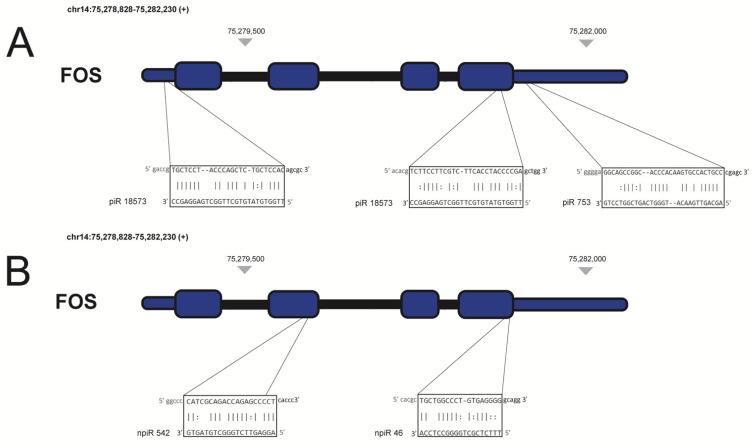
Known and novel piRNA targeting *FOS* mRNA transcript. piRNAs were shown to be differentially expressed during 1 and 2 h after *Trypanosoma cruzi* challenge of primary human cardiac myocytes. piRNAs are mapped to specific genic regions based on complementarity of piRNA/mRNA sequences using miRANDA and RNA22 software packages. Exons are represented by blocks, while grey lines represent intronic regions. Thinner blocks represent 5′ and 3′ untranslated regions (UTRs) at the terminus of *FOS* gene. (**A**) Known piRNAs upregulated at 1-h post infection (**B**) novel piRNAs upregulated at both 1 and 2 h post infection.

*Trypanosoma cruzi*, the causative agent of Chagas heart disease, can infect all nucleated cells of the body. During the process of infection, the parasite induces significant changes in the gene expression profiles of the host cells [37,151,152,153] through unknown mechanisms. To elucidate the mechanisms by which the parasite dysregulates host gene expression profile during the early phase of infection, our group challenged primary human cardiomyocytes with the parasite for 1 and 2 h, and purified the small RNAs for RNA-sequencing. We observed that 207 known and novel piRNAs were dysregulated after 1- and 2-h following *T. cruzi* challenge. Though hundreds of piRNAs were dysregulated during the early phase of infection, we were interested in the piRNAs that can target mRNAs that could be important in *T. cruzi* infection and pathogenesis. Our analysis showed that some dysregulated novel piRNAs targeted TGFβ, a known profibrotic cytokine that is upregulated during *T. cruzi* infection and suggested to play a role in *T. cruzi* induced fibrotic pathophysiology [154,155,156]. FOS, a transcription factor belonging to the AP-1 family, is another profibrotic gene that is upregulated during *T. cruzi* infection [153]. Our analysis showed that differentially expressed novel and known piRNAs including piR-753 and piR-18573 mapped to the transcript of FOS (Figure 2).

Furthermore, 5 known piRNAs were shown to target NFACT2, a known pro-inflammatory transcription factor that has been shown to play a role in the pathobiology of cardiomyopathy [157,158,159]. We implicated several piRNAs as potential modulators of genes influencing parasite infectivity and pathogenesis. Functional studies and characterization of these novel piRNAs reported by our group will strengthen the role of piRNAs in *T. cruzi* pathogenesis. Evaluation of piRNA expression profiles in the context of host-parasite interactions has the potential to delineate their molecular functions and pathophysiological importance during infection and pathogen induced pathogenesis [37].

## 7. Conclusions

piRNAs and PIWI proteins have been regarded as guardians of the germline, as they protect the genome from TEs. Accumulating evidence denotes piRNAs as vital regulators of important cellular processes conserved among various organisms. This has led investigators to consider potential noncanonical roles of piRNAs. The mechanisms through which piRNAs contribute to various disease pathogenesis remain unknown and elusive. However, studies are consistently implicating various important roles for piRNAs outside of the germline. Further characterization of piRNA function is required to understand how these sncRNAs regulate gene expression in a multitude of circumstances within the host. Limitations of piRNA validation can be overcome with high quality RNA-sequencing approaches. As our understanding of piRNA biology and function increases, so will the availability of assays/experiments to uncover the exact mechanisms of piRNA function; how many piRNAs targeting a gene of interest are required to carry out a specific function, how are piRNAs targeting genes that function differently in different conditions regulated. These types of conceptual studies will provide critical insights into piRNAs as disease biomarkers and modulators of gene expression.

## Figures and Tables

**Table 1:** piRNAs associated with various diseases.

Disease/ Pathology	piRNA	Upregulated/ Downregulated	Gene Target	Reference
Cardiac Hypertrophy/Cardiac Failure	piR-141981/cardiac-hypertrophy-associated piRNA (CHAPIR)	Upregulated in mice that underwent transverse aortic construction surgery	*Myh7*, *Anp*, *Bnp*, and PARP10 mRNA	[22]
Cardiac Hypertrophy/Cardiac Failure	piR-13375 and piR-106654	Downregulated in mice that underwent transverse aortic construction surgery	Not identified	[22]
Cardiac Failure	has-piR-006426 and has-piR-0200009	Downregulated in the serum exosomes of patients experiencing heart failure	Not identified	[23]
Myocardial Infarction	piR_2106027	Upregulated in the serum of patients with Troponin-I negative myocardial infarction	Troponin-I	[24]
Cardiac Tissue Regeneration/ Angiogenesis	piR-55490	Not specified	*mTOR*	[25,26]
Breast Cancer	piR-4987	Upregulation promotes lymph node metastasis	Not identified	[27]
Breast Cancer	piR-021285	Not specified	Induces methylation several genes related to breast cancer in vitro	[28]
Lung Cancer	piR-L-163	Upregulated upon cisplatin treatment in vitro and in vivo	Regulates ezrin-radixin-moesin (ERM) proteins	[29]
Lung Cancer	piR-651	Upregulated	Promotes the upregulation of cyclins and CDKs	[30]
Colorectal Cancer	piR-1245	Upregulation promotes colorectal cancer tumor growth	Suppresses multiple tumor suppressors such as ATF3, DUSP1, and SESN2	[31]
Gastric Cancer	PiRNA-823	Upregulated in patients with multiple myeloma	Regulates de novo DNA methylases DNMT3A and 3B and angiogenesis	[32]
Bladder Cancer	piRNA-DQ594040	Upregulated in tissue from patients with bladder cancer	May regulate Tumor Necrosis Factor Superfamily Member 4 (TNFSF4)	[33]
Liver Cancer	piR-Hep1	Upregulated in hepatocellular carcinoma samples	Upregulates PI3K/AKT	[34,35]
Pancreatic Cancer	piR-017061	Downregulated in pancreatic ductal adenocarcinoma tissue	Not identified	[36]
Pancreatic Cancer	piR-016658 and piR-001311	Have been detected in the extracellular RNA of pancreatic cancer patients	Not identified	[36]
*Trypanosoma cruzi* infection in cardiac myocytes	piR-753 and piR-18573	Upregulate d during early phase of infection	FOS	[37]
